# Modified vein clamping technique for renal cell carcinoma complicated with level I–II IVC thrombi: a study at a single centre

**DOI:** 10.1186/s12894-021-00947-9

**Published:** 2021-12-21

**Authors:** Jiaxing Ma, Wei Sun, Weiwei Qian, Jie Min, Tao Zhang, Dexin Yu

**Affiliations:** Department of Urology, The Second Affiliated Hospital of Anhui Medical University, 678 Furong Rd, Hefei, 230032 Anhui People’s Republic of China

**Keywords:** Renal cell carcinoma, Tumor thrombus, Laparoscopy, Modified vein clamping technique

## Abstract

**Objectives:**

To share our initial experience with the modified vein clamping technique for the treatment of renal cell carcinoma complicated with level I–II IVC thrombi.

**Methods:**

From March 2018 to April 2021, 11 patients with renal cell carcinoma (RCC) involving an IVC tumour thrombus were admitted to our hospital. They all underwent laparoscopic radical nephrectomy and IVC thrombectomy (LRN-IVCTE) using a modified vein clamping technique.

**Results:**

All procedures were successfully completed without conversion to open surgery. The median operative time was 185.00 min (145.00–216.00 min); the median estimated blood loss was 200.00 ml (155.00–300.00 ml), and four patients received an intraoperative transfusion. In addition, the median IVC clamping time was 18.00 min (12.00–20.00 min); the median postoperative hospital stay was 6.00 days (4.00–7.00 days), while the median follow-up period was 28.00 months (4.00–34.00 months).

**Conclusions:**

The modified vein clamping technique for the treatment of renal cell carcinoma complicated with level I–II IVC thrombi may be a safe and technically feasible alternative technique.

## Introduction

Tumour thrombi can be found in many cases of renal cell carcinoma (RCC). Most tumour thrombi are located in the renal vein and inferior vena cava, accounting for approximately 10%, while a few are located in the heart, accounting for approximately 1% [1]. Compared with other treatments, nephrectomy with IVC thrombectomy is becoming a more popular option because it provides a better prognosis [2]. For the treatment of RCC, patients who underwent radical nephrectomy with any level of IVC thrombectomy had a satisfactory 5-year survival rate of 64% [3]. In fact, with the introduction of novel therapeutic agents, the survival rate has significantly improved recently. Regardless of the thrombus level, early surgical resection is still the classic treatment in nonmetastatic RCC patients with an IVC thrombus [4]. In addition, in metastatic RCC patients, treatment of the primary tumour may be indicated in the cytoreductive setting, as it ensures a lower level of circulating tumour-derived immunosuppressive factors and a better prognosis [5]. Furthermore, the open approach has previously been considered the standard when performing radical nephrectomy with IVC thrombectomy. More recently, the focus has shifted towards minimally invasive techniques (laparoscopy and robotic assistance), whose feasibility for performing extremely complex surgeries has been proven [6]. In this case, ensuring maximal oncologic control with minimal morbidity for the patient is the current challenge for the surgical treatment of RCC with an IVC thrombus. Since Varkarakis et al. [7] first reported their experience with LRN-VCTE in 2004, several reports have been published [6, 8–10]. Recently, robot-assisted surgery has also been reported [11–13]. All these reports have only described their major surgical procedure and oncologic results, whereas there is no relevant research on the technique of vein clamping.

In this paper, we present our initial experience with a modified vein clamping technique for the treatment of renal cell carcinoma with a level I–II IVC thrombus at our single medical centre.

## Patients and methods

### Patients

We retrospectively evaluated the records of 11 patients with RCC involving an IVC tumour thrombus (level I–II) who underwent laparoscopic radical nephrectomy and IVC thrombectomy (LRN-IVCTE) at our hospital from March 2018 to April 2021. However, patients in whom the primary tumour was invading adjacent organs, who had multiple distant metastases, or in whom the IVC was extensively infiltrated by the thrombus were excluded from laparoscopic IVC thrombectomy, except for the single metastatic lesion. Moreover, patients with a history of upper abdominal surgery and those with an unacceptable anaesthetic risk and cardiopulmonary insufficiency were also not included. Patient characteristics (age, sex, body mass index, KPS score, ASA score, clinical stage, renal tumour size, IVC thrombus classification, and thrombus length) were assessed. In total, eight cases had RCC on the right side, and three had RCC on the left. All patients underwent abdominal magnetic resonance imaging (MRI), abdominal enhanced computed tomography (CT), and chest CT before the operation so that the tumour size (cm) and thrombus length (cm) were measured from CT or MRI. In addition, through renal emission computed tomography (ECT), it was determined that all patients had a normal kidney on the contralateral side, whereas the Mayo classification was used to evaluate the position of the IVC thrombus [14], with the levels defined as follows: level 0, a thrombus limited to the renal vein; level I, a tumour thrombus extending ≤ 2 cm above the renal vein; level II, an extension of > 2 cm above the renal vein, but below the hepatic vein; level III, a thrombus at the level of or above the hepatic vein but below the diaphragm; and level IV, extension above the diaphragm or into the right atrium. Here, it should be mentioned that two patients with single metastasis in the lung were administered preoperative neoadjuvant targeted therapy for 3 months. RCC was classified according to the American Joint Committee on Cancer 2010 TNM staging criteria [15].

Perioperative data (median operative time, estimated blood loss, IVC clamping time, lymphadenectomy surgical time, blood transfusion, preoperative and postoperative serum creatinine, preoperative and postoperative alanine aminotransferase (ALT) and aspartate aminotransferase (AST), preoperative and postoperative haemoglobin, and perioperative complications) were assessed. In addition, perioperative complications were graded according to the Clavien–Dindo classification [16].

All procedures were performed by a single surgeon (Dexin Yu) with LRN-IVCTE using a modified vein clamping technique.

This study was carried out in accordance with the Helsinki Declaration and was approved by the Research Ethics Committee at the Second Affiliated Hospital of Anhui Medical University. In addition, written informed consent was obtained from all participants prior to their inclusion within this study.

### Preoperative preparation

Following the enhanced recovery after surgery (ERAS) protocol, general preoperative preparation included preoperative skin preparation, fasting for 6 h and water deprivation for 2 h, except for a water enema and placement of an indwelling gastric tube. The anticoagulation therapy used in these patients was 1 mg/kg enoxaparin administered subcutaneously twice a day from the moment of diagnosis and paused 12 h before surgery. Then, twelve hours after the procedure, anticoagulation therapy was resumed and continued for up to 21 days [17, 18]. Moreover, special preoperative preparation included renal artery embolization on the related side 1–2 h before the operation in 3 patients who were diagnosed with RCC on the left side with an IVC tumour thrombus.

### Surgical procedure

The pure LRN-IVCTE was performed in all cases. All procedures were followed by a single surgical team with experience in open surgery and laparoscopic surgery, and the transperitoneal approach was adopted in all patients. Furthermore, transoesophageal echocardiography was used to monitor the extent and stability of the thrombus and to ensure that the tumour thrombus was removed completely during surgical manipulation. Extension of lymphadenectomy should involve the lymph nodes surrounding the ipsilateral great vessel and the inter-aortocaval region from the crus of the diaphragm to the inferior mesenteric artery.

**Fig. 1:**
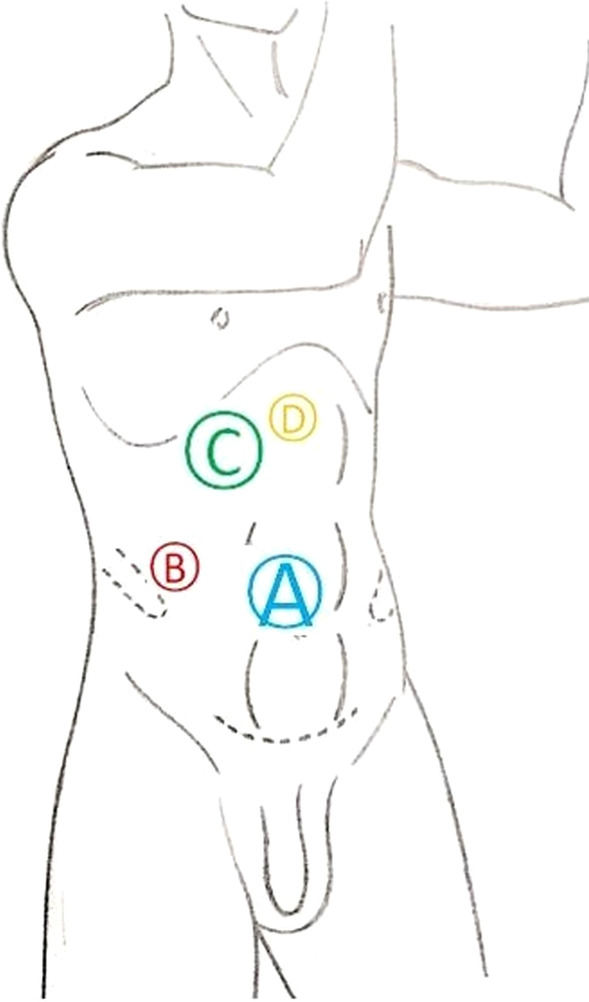
The positioning of the trocars for the transperitoneal approach: the optic trocar (10 mm) lateral to the umbilicus, at the lateral border of the abdominal rectus muscle (**A**); the trocar for the left-hand instrument on the axillary line parallel to the navel (**B**); the trocar for the right-hand instrument (12 mm) on the perpendicular line from the umbilicus to the costal margin, 2 cm below the costal margin (**C**); a 4th trocar (5 mm) for the assistant to use for liver retraction and suction in the epigastric region (**D**)

**Fig. 2:**
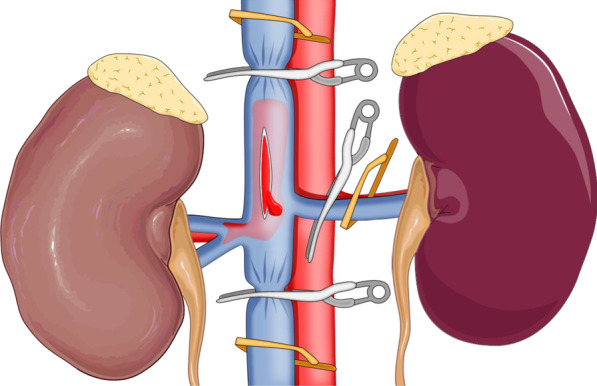
The caudal IVC, left renal vein, and cephalic IVC were sequentially clamped using laparoscopic bulldog clamps. We used laparoscopic bulldog clamps to clamp the veins by moderately pulling the vessel loops and narrowing the venous wall

**Fig. 3:**
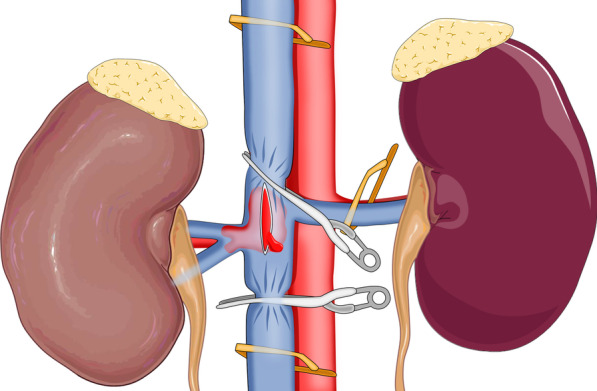
For level I IVC thrombi, the caudal IVC was clamped first, and then we used just one laparoscopic bulldog clamp to clamp the left renal vein and cephalic IVC at the same time

For right RCC, no patient accepted preoperative right renal artery embolization. After general anaesthesia and Foley catheter placement, patients were placed in the 70° flank position on a flat bed, when four laparoscopic ports were in the right lumbar area (Fig. 1). In addition, insufflation with CO2 at a pressure of 15 mmHg was conducted, the hepatocolic ligament was incised, and the liver was retracted cephalically. After mobilization of the colon and duodenum, the IVC was frontally exposed. Then, the surfaces of the right and left renal veins were isolated. The IVC was mobilized above and below the renal vein for a distance of 3–5 cm along the length of the thrombus, and the lumbar veins were transected. For level II IVC thrombi, the gonadal vein and accessory hepatic veins were also clipped and divided for circumferential dissection of the IVC. Next, the right artery was exposed and ligated between the IVC and aorta ventralis. Then, the vessel loops were placed under the IVC above and below the thrombus and around the left renal vein, which was secured with a Hem-o-lok clip prepared for clamping, followed by sequential clamping of the caudal IVC, left renal vein, and cephalic IVC with laparoscopic bulldog clamps. Here, it should be noted that laparoscopic bulldog clamps rather than the vessel loops directly with Hem-o-lok clips were used to clamp the veins by moderately pulling the vessel loops and narrowing the venous wall. After occlusion of the above vessels, the IVC wall was incised at the right renal vein ostium to avoid stenosis after suturing the inferior vena cava (Fig. 2). Then, the thrombus was removed and fully covered with a specimen bag to prevent tumour dissemination. After the IVC lumen was irrigated with heparinized saline, the IVC was repaired with a continuous suture using the 5–0 polypropylene suture. Before the IVC was closed, the IVC tourniquet was loosened to remove any clot in the IVC. After that, the right kidney was subsequently mobilized, excised, and placed with the main body of the thrombus into the specimen bag, followed by removal through the abdominal incision. In addition, for the level I IVC thrombus, first, the caudal IVC was clamped; then, only one laparoscopic bulldog clamp was employed to clamp the left renal vein and the cephalic IVC simultaneously (Fig. 3).

For left RCC, all three patients underwent preoperative left renal artery embolization so that the IVC thrombus could be directly handled when the position and placement of ports were the same as those for right RCC (Fig. 1). The IVC combined with the right and left renal veins was isolated sequentially. Beyond that, the caudal IVC, right renal vein, and cephalic IVC were sequentially clamped using laparoscopic bulldog clamps, while the IVC wall was incised at the left renal vein ostium. In the same way, a specimen bag was used to cover the thrombus to avoid tumour dissemination. In general, the specimen bag was seamed with silk thread. After this procedure, the patients were converted to a right lateral decubitus position, and left LRN was performed.

## Result

As shown in Table 1, the median age was 57.00 years (54.00–71.00 years); ten of eleven patients were men; the median tumour size was 7.20 cm (6.00–10.50 cm), and eight of eleven patients were on the right side; the median thrombus length was 3.00 cm (2.00–4.00 cm). Six patients had a level I thrombus, while five had a level II thrombus. We identified two patients with a single metastasis in the lung who were administered preoperative neoadjuvant targeted therapy. One patient did not experience tumour or thrombus shrinkage before nephrectomy. One patient experienced tumour shrinkage (shrinkage from 15.1 cm to 13.2 cm) and thrombus shrinkage (shrinkage from 6.0 cm to 4.5 cm) preoperatively. The IMDC scores for the two metastatic patients were both 2. No patient was converted to open surgery.

**Table 1:** Baseline characteristics of all 11 patients

Characteristics	Value
Patients, n	11
Gender, n (%)	
Male	10 (90.9)
Female	1 (9.1)
Median age, years (IQR)	57.00 (54.00–71.00)
Median BMI, kg/m^2^ (IQR)	21.36 (20.42–25.21)
Median KPS score (IQR)	90.00 (80.00–90.00)
ASA score, n (%)	
II	7 (63.6)
III	4 (36.4)
Affected side, n (%)	
Left	3 (27.3)
Right	8 (72.7)
Median tumor size, cm (IQR)	7.20 (6.00–10.50)
Clinical stage, n (%)	
T3bN0M0	9 (81.8)
T3bN1M0	0 (0)
T3bN0M1	2 (18.2)
IVC thrombus level, n (%)	
I	6
II	5
Median IVC thrombus length, cm (IQR)	3.00 (2.00–4.00)
Preoperative embolization, n (%)	
Yes	3 (27.3)
No	8 (72.7)

Continuous data are reported as median (IQR)

*BMI* body mass index, *KPS* Karnofsky Performance Status, *ASA* American Society of Anesthesiologists, *IVC* inferior vena cava, *IQR* interquartile range

The perioperative data are shown in Table 2. The median operative time was 185.00 min (145.00–216.00 min); the median estimated blood loss was 200.00 ml (155.00–300.00 ml). Four patients received an intraoperative transfusion. The transfusion was due to preoperative anaemia in three of the patients. The fourth patient’s case was complicated with coronary heart disease, while intraoperative arterial blood gas analysis of haemoglobin was 88 g/l. Considering that blood loss would affect the heart blood supply, blood transfusion was given to improve the heart blood supply and avoid increasing the burden on the heart after discussion with the anaesthesiologist. The median IVC clamping time was 18.00 min (12.00–20.00 min), while the lymphadenectomy surgical time was 22.00 min (20.00–24.00 min). The median postoperative hospital stay was 6.00 days (4.00–7.00 days). Moreover, histological analysis revealed clear cell carcinoma in all patients. Surgical margins were negative in all cases, and no major complications were noted (Clavien–Dindo grade ≥ 3). All cases were pathologically confirmed to be renal clear cell carcinoma without regional lymph node metastasis.

**Table 2:** Perioperative data

Characteristics	Value
Median operative time, min (IQR)	185.00 (145.00–216.00)
Median IVC clamping time, min (IQR)	18.00 (12.00–20.00)
Median lymphadenectomy surgical time, min (IQR)	22.00 (20.00–24.00)
Median estimated blood loss, ml (IQR)	200.00 (155.00–300.00)
Patients receiving transfusion, n (%)	4 (36.4)
Median day to surgical drain removal, day (IQR)	5.00 (3.00–6.00)
Median day to full ambulation, day (IQR)	2.00 (1.00–2.00)
Median day to oral feeding, day (IQR)	2.00 (1.00–2.00)
Median postoperative hospital stay, day (IQR)	6.00 (4.00–7.00)
Perioperative complications, n (%)	
Low grade Clavien I–II	4 (36.4)
High grade Clavien III–IV	0 (0)
Median follow-up, month (IQR)	28.00 (4.00–34.00)

Continuous data are reported as median (IQR)

*IVC* inferior vena cava, *IQR* interquartile range

Preoperative and postoperative data are shown in Table 3. The median and interquartile range for all continuous variables were used, while we used the Wilcoxon signed ranks test to compare preoperative and postoperative data. All patients exhibited mild renal insufficiency. No significant difference was found in AST between preoperation and postoperation measurements. Significant differences were found in haemoglobin and ALT between preoperation and postoperation measurements. However, the value of postoperative ALT was within the normal range. The median follow-up period was 28.00 months (4.00–34.00 months). In addition, pulmonary metastasis was identified before surgery in two patients who survived and were treated with targeted therapy (pazopanib), whereas the remaining nine patients had no local recurrence or distant metastasis during follow-up.

**Table 3:** Preoperative and postoperative data

Characteristics	Preoperative	Postoperative	Z value	P value
Median SCr, μmol/l (IQR)	79.00 (75.00–91.00)	110.00 (79.00–148.00)	− 2.578	0.010
Median hemoglobin, g/l (IQR)	123.00 (96.00–134.00)	100.00 (93.00–110.00)	− 2.492	0.013
Median AST, μ/l (IQR)	23.00 (17.00–32.00)	25.00 (21.00–31.00)	− 1.946	0.052
Median ALT, μ/l (IQR)	18.00 (12.00–29.00)	25.00 (15.00–29.00)	− 2.492	0.005

Continuous data are reported as median (IQR)

*SCr* serum creatinine, *AST* aspartate aminotransferase, *ALT* alanine aminotransferase, *IQR* interquartile range

## Discussion

Traditionally, the classic surgical approach of radical nephrectomy and IVC thrombectomy is open surgery. For RCC patients with an IVC thrombus, radical nephrectomy with thrombectomy can provide a better prognosis than nonaggressive surgical treatment [2]. Therefore, surgery is a priority for patients with suitable physical conditions. Moreover, with the development of surgical techniques and perioperative management, pure laparoscopic and robotic-assisted thrombectomy has become safe and feasible [8]. Currently, in most reports, vessel loops are used to clamp veins [19, 20], but we believe vein injury would occur if the vessel loops are pulled tautly, and this procedure cannot ensure successful clamping of the vein on one attempt. In addition, the vessel should be wrapped twice, which may prolong the operation time. Furthermore, surgeons have used a Hem-O-lok to tighten the vessel loops, indicating that they need to cut off the Hem-O-lok when loosening the block, which is less convenient than the laparoscopic bulldog clamp. Shao et al. [8] reported their experience in using open bulldog clamps to clamp renal veins and the IVC successfully in laparoscopic surgery, but it is known that using a laparoscopic instrument to control open bulldog clamps is not convenient and that the bulldog clamps can slide out of the laparoscopic instrument during the clamping procedure. In our single medical centre, the vessel loops were combined with laparoscopic bulldog clamps to clamp veins as described.

During minimal access surgery, small tricks may yield important gains. In this report, the application of laparoscopic bulldog clamps in laparoscopic urologic surgery was presented. In fact, the use of bulldog clamps during laparoscopic or robotic procedures, especially in urology surgery, is common (such as in laparoscopic or robotic partial nephrectomy). Specifically, bulldog clamps were used to clamp the renal artery before excising the renal mass. Then, it was proven that the clamps can thoroughly clasp the renal artery. In this report, laparoscopic bulldog clamps combined with vessel loops were used to clamp the caudal IVC, left renal vein and cephalic IVC. By moderately pulling the vessel loops and narrowing the venous wall, the veins could be clamped using laparoscopic bulldog clamps easily and conveniently. After early control of the renal artery and the lumbar veins to reduce haemorrhagic complications, it is believed that the use of laparoscopic bulldog clamps for the occlusion of the vessels during IVC thrombectomy may be preferable to the tourniquets or vessel loops, since slow retrograde bleeding up to the end of IVC suturing was experienced. Using only vessel loops is time-consuming, and cases may be complicated with caval injury if force is inequitably applied to block veins. However, laparoscopic bulldog clamps are easy to use with the aid of an applicator. In addition, this approach is safe, quick and reproducible while providing good surgical exposure in a limited surgical field without interfering with the laparoscopic instrument and blocking any trocar access during the operation. Moreover, the bulldog clip always exerts the same defined pressure, minimizing the damage to the veins. Otherwise, in cases of a level I IVC thrombus, the thrombus was milked down, followed by clamping of the caudal IVC. Then, the vessel loops combined with contralateral renal veins and the cephalic IVC were moderately pulled to narrow the venous wall. To make the next clamping step easy, the direction in which vessel loops are pulled should be adjusted properly. The angle between the inferior vena cava and the left renal vein should be narrowed to an acute angle as far as possible by proper pulling of the vessel loops to reduce the distance between the inferior vena cava and the left renal vein. In particular, attention should be given to the force exerted in pulling the vessel loops in order to avoid vein injury. Next, one laparoscopic bulldog clamp is adopted to clamp the left renal vein and cephalic IVC at the same time, thus saving operative time. Laparoscopic bulldog forceps are easy to use with the aid of a companion applicator. This set of instruments has been routinely used in laparoscopic partial nephrectomy.

The reported operative time when performing IVC thrombectomy ranged between 100 and 275 min, the IVC clamping time varied between 12 and 25 min, and the mean blood loss measured between 150 and 320 ml [8, 19], which was similar to our experience. In addition, none of the authors pointed out significant intraoperative or postoperative complications or conversions to open surgery, and the patients were discharged 9 days after the operation [8], which is similar to our figures to some extent. All of the cases we reported successfully completed surgery without serious complications. All the instruments used in our operation are conventional laparoscopic instruments, which are easily available in all centres. It has good feasibility and popularity.

In addition, to ensure that laparoscopic thrombectomy can be performed safely, patients need to be carefully selected. The criteria for a qualified patient are as follows: 1. the primary tumour is localized; 2. the intraluminal thrombus is free floating, without extensive involvement of the peripheral tissue or vascular wall [8]. Open surgery may be considered if the thrombus appears to be fixed or if the IVC wall is extensively involved [21].

Early control of the renal artery before renal vein manipulation and kidney mobilization is essential to minimize surgical risks, as this helps to decrease intraoperative bleeding and prevent thrombus detachment caused by venous blood flow. In addition, it can also reduce blood flow, with the kidney shrinking, allowing for easier handling, while the thrombus may also shrink. Wang et al. [19] reported that they underwent special preoperative preparation including renal artery embolization on the related side 1–2 h before the operation. However, we think renal artery embolization is unnecessary for right RCC because the right artery can be easily exposed and ligated between the IVC and aorta ventralis. As the surgical procedure was described, when the IVC and the left renal vein were exposed, the right renal artery in the interaortocaval space could be easily found before clamping the IVC. For left RCC, it is believed here that renal artery embolization is necessary. Chopra et al. carried out renal artery embolism before surgery in 80.3% of their patients [13]. It is difficult to expose the left renal artery when the IVC thrombus is handled in the left decubitus position. For left-side tumour cases, the procedures for pedicle control and kidney mobilization require operation from the left side, while major vessel control and thrombectomy should be operated on from the right side, which is more complex than right-side cases. Thus, left renal artery embolization before surgery leads to decompression of venous collaterals and decreased blood loss [22]. In this case, this technology can reduce the difficulty of surgery.

Preventing the risk of pulmonary embolism as a result of thrombosis during the procedures is one of the most important tips. Chopra et al. proposed the “IVC-first, kidney-last” technique for robot-assisted IVC thrombectomy [13]. He detached the tumour thrombus using a stapler to separate the part of the tumour thrombus from that of nephrectomy, but the tumour thrombus may shatter toward the cephalad side at the moment when pressure with the stapler is applied to the renal vein. In addition, the vein wall might collapse, and the tumour thrombus might be seeded into the peritoneal cavity. Yoichiro T et al. adopted a method of “en bloc” nephrectomy with IVC thrombectomy, where detaching the tumour thrombus is unnecessary [23]. However, that method also features two main disadvantages. Specifically, one is that a twisting force is applied to the renal vein (containing the tumour) during kidney mobilization, which may cause the tumour thrombus to unexpectedly break and scatter, while the other is difficulty in mobilizing the kidney in cases where the tumour is large, particularly when the renal vein is not detached and adheres to the IVC. In our single centre, for right RCC, the modified “IVC-first, kidney-last” technique was proposed. After artery control, the IVC tumour thrombus still attached to the renal vein was removed, and then, the tumour thrombus was placed in a specimen bag that was closed immediately by using 2–0 silk. After kidney mobilization, the kidney combined with the tumour thrombus was placed in a larger specimen bag.

The present study has several limitations. First, it was a single-centre study with a small patient population. Second, there are few reports on long-term oncological outcomes, and further research is required. Third, further animal studies are required to verify the difference between laparoscopic bulldog clamps and vessel loops that block veins. However, given the lack of studies on using laparoscopic bulldog clamps in IVC tumour thrombi, it is believed that the present study has clinical significance.

## Conclusions

The modified vein clamping technique for the treatment of renal cell carcinoma complicated with a level I–II IVC thrombus is a valid alternative. However, larger patient samples and longer follow-up times are required to further define the role of modified laparoscopic surgery for high-level IVC thrombi in RCC patients.

## Data Availability

The datasets used and/or analysed during the current study are available from the corresponding author on reasonable request.
